# Supplementary material for: Fyn Phosphorylates Transglutaminase 2 (Tgm2) and Modulates Autophagy and p53 Expression in the Development of Diabetic Kidney Disease
Source: Cells. 2023 Apr 20;12(8):1197. doi: 10.3390/cells12081197 (PMC10136856; doi:10.3390/cells12081197)
Supplement: Supplementary file 1 [file cells-12-01197-s001.zip › cells-2239541-supplementary.pdf]

Supplementary Material

A

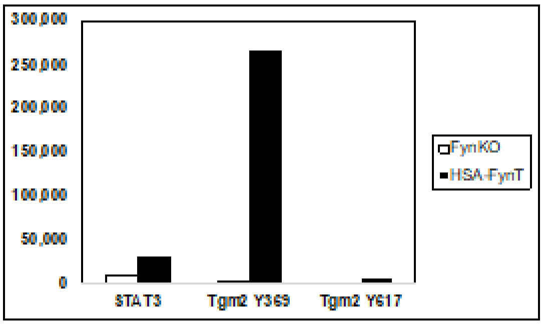

B

Protein Name	Site	Peptide	HSA-FynT #1	HSA-FynT #2	Fyn KO #1	Fyn KO #2
STAT3	Y705	YCRPESQEHPEADPG SAAPY*LK	27,205	33,603	10,586	8,363
Tgm2	Y369	SEGTY*CCGPVSVR	301,653	227,355	0	3,388
Tgm2	Y617	LVAEVSLKNPLSDPLY *DCIFVEGAGLTK	6,066	5,087	0	0

C

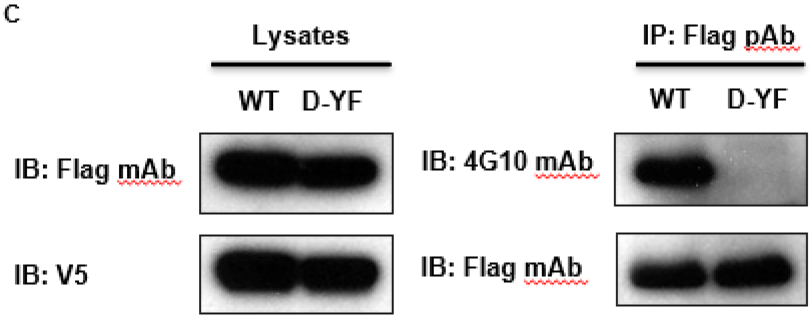

Figure S1. (A,B) Tyrosine PhosphoScan® Proteomics screening was provided by Cell signaling Technology (Boston, MA) as detailed on their website: https://www.cellsignal.com/services/proteomics-analytical-services/ptmscan-discovery-proteomic-services. Briefly, gastrocnemius skeletal muscle extracts of Fyn null mice and the HSA-FynT mice were digested with trypsin followed by immunoprecipitation with the phosphotyrosine PY100 antibody. The immunoprecipitates were then subjected to LC-MS/MS analysis to identify tyrosine-phosphorylated proteins. **(C)** 48 h after HEK-293T were co-transfected with V5-Fyn-CA and Flag-Tgm2 or Flag-Tgm2-Y369/617F, immunoprecipitation was performed using a Flag antibody followed by western blotting with the indicated antibodies. Blots are representative images of three independent experiments.

A

Mouse Tyrosine predictions

# Tyr	Context	Score	Kinase
44	RLTL**Y**FEGR	0.373	SRC
50	EGRG**Y**EASV	0.486	SRC
115	PIGL**Y**RLSL	0.327	SRC
125	ASTG**Y**QGSS	0.388	SRC
139	FILL**Y**NAWC	0.350	SRC
149	ADDV**Y**LDSE	0.493	SRC
159	ERRE**Y**VLTQ	0.437	SRC
168	QGFI**Y**QGSV	0.356	SRC
219	SSPI**Y**VGRV	0.393	SRC
245	WDNN**Y**GDGI	0.426	SRC
274	QQVK**Y**GQCW	0.357	SRC
301	VVTN**Y**NSAH	0.375	SRC
315	LLIE**Y**FRNE	0.394	SRC
351	LQPG**Y**EGWQ	0.402	SRC
**369**	SEGT**Y**CCGP	**0.517**	SRC
388	LSTK**Y**DAPF	0.410	SRC
443	ITHT**Y**KYPE	0.429	SRC
445	HTYK**Y**PEGS	0.318	SRC
516	RTVS**Y**NGVL	0.328	SRC
536	TLDP**Y**SENS	0.418	SRC
547	LRIL**Y**EKYS	0.380	SRC
550	LYEK**Y**SGCL	0.314	SRC
574	AANS**Y**LLAE	0.344	SRC
582	ERDL**Y**LENP	0.441	SRC
617	SDPL**Y**DCIF	0.481	SRC
678	SVKG**Y**RNVI	0.326	SRC

B

human Tyrosine predictions

# Tyr	Context	Score	Kinase
50	EGRN**Y**EASV	0.480	SRC
115	PIGL**Y**RLSL	0.327	SRC
125	ASTG**Y**QGSS	0.388	SRC
149	ADAV**Y**LDSE	0.425	SRC
159	ERQE**Y**VLTQ	0.438	SRC
168	QGFI**Y**QGSA	0.358	SRC
219	SSPV**Y**VGRV	0.413	SRC
245	WDNN**Y**GDGV	0.430	SRC
274	QRVK**Y**GQCW	0.338	SRC
301	VVTN**Y**NSAH	0.375	SRC
315	LLIE**Y**FRNE	0.394	SRC
351	LQPG**Y**EGWQ	0.412	SRC
**369**	SEGT**Y**CCGP	**0.504**	SRC
388	LSTK**Y**DAPF	0.410	SRC
443	ITHT**Y**KYPE	0.429	SRC
445	HTYK**Y**PEGS	0.317	SRC
503	TAEE**Y**VCRL	0.376	SRC
516	RTVS**Y**NGIL	0.317	SRC
528	CGTK**Y**LLNL	0.319	SRC
548	LCIL**Y**EKYR	0.381	SRC
551	LYEK**Y**RDCL	0.308	SRC
575	VINS**Y**LLAE	0.362	SRC
583	ERDL**Y**LENP	0.441	SRC

Figure S2. The result of potential tyrosine phosphorylation sites for Tgm2 predicted by Netphos3.1 software: https://services.healthtech.dtu.dk/service.php?NetPhos-3.1 for mouse (**A**) and human (**B**). Accessed 20 July 2022.

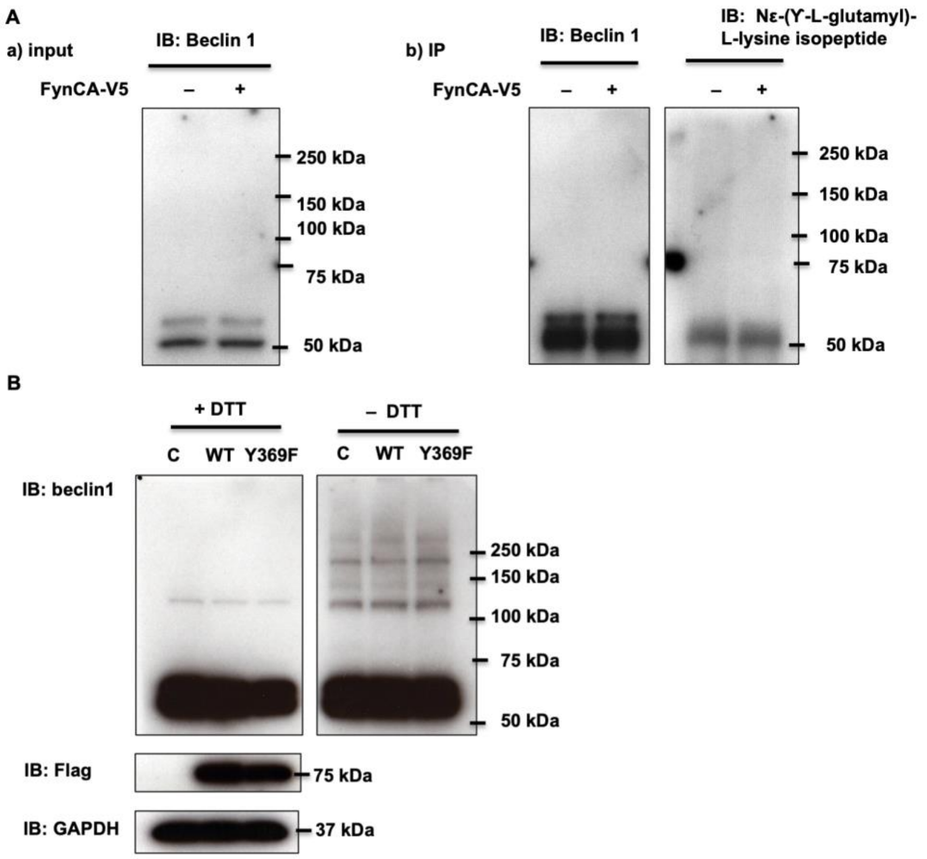

Figure S3. (A) 48 h after HEK-293T were co-transfected with V5-Fyn-CA, Beclin 1 immunoprecipitation was performed using a Beclin 1 antibody followed by western blotting for indicated antibodies. Blots are representative images of three independent experiments. **(B)** 48 h after HEK-293T were co-transfected with Flag-Tgm2 or Flag-Tgm2-Y369F, immunoblots was performed using either denatured (+DTT) or non-denatured (–DTT) condition by western blotting with the indicated antibodies. Blots are representative images of three independent experiments.

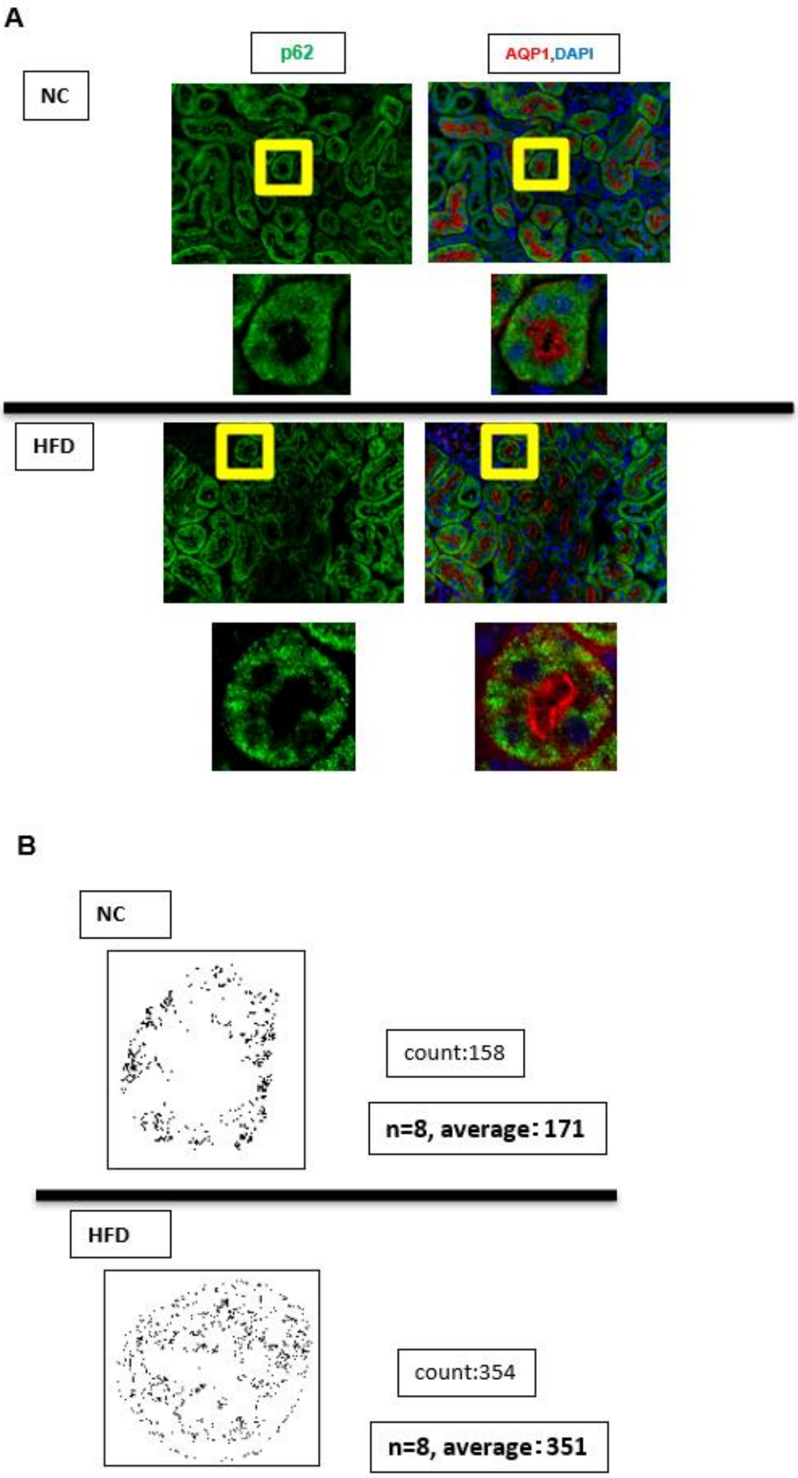

Figure S4. (A,B) Representative images and green dots for counting p62 puncta. The yellow boxed image was shown enlarged below.

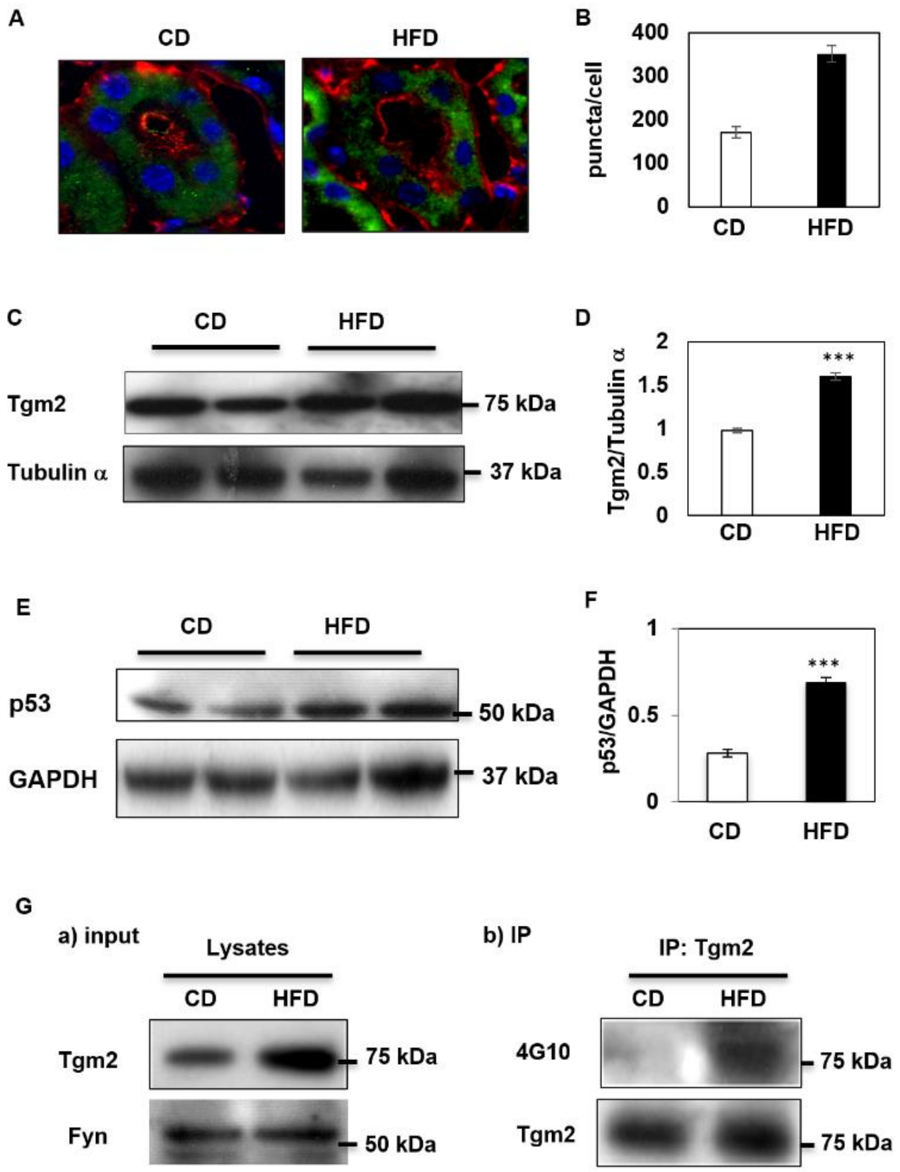

Figure S5. (A,B) p62 punta in kidneys were visualized and quantified in the kidneys of standard diet fed (CD) and high fat diet-fed (HFD) control mice. **(C)** Control mice were fed a standard diet (CD) or a high fat diet (HFD) (60% kcal) for 12 weeks. Fyn, Tgm2 protein levels were measured using immunoblotting and normalized to tubulin α levels **(D). (E)** p53 protein expression was measured by immunoblotting in kidneys of control mice fed either a standard diet (CD) or a high fat diet (HFD) and normalized against GAPDH levels **(F)**. **(G,H)** Tgm2 was immunoprecipitated and phosphorylation levels were assessed. Data are shown as the mean ± standard error of the mean (s.e.m.), *** $p < 0.001$.
